# Resting Heart Rate Variability Predicts Vulnerability to Pharmacologically-Induced Ventricular Arrhythmias in Male Rats

**DOI:** 10.3390/jcm8050655

**Published:** 2019-05-10

**Authors:** Luca Carnevali, Rosario Statello, Andrea Sgoifo

**Affiliations:** Stress Physiology Lab, Department of Chemistry, Life Sciences and Environmental Sustainability, University of Parma, 43121 Parma, Italy; luca.carnevali@unipr.it (L.C.); rosarioignazio.statello@studenti.unipr.it (R.S.)

**Keywords:** arrhythmias, heart rate variability, rats, sudden death, vagal tone

## Abstract

The electrical stability of the myocardium is dependent on the dynamic balance between sympathetic and parasympathetic influences on the heart, which is reflected by heart rate variability (HRV). Reduced HRV is a proposed predictor of sudden death caused by ventricular tachyarrhythmias in cardiac patients. However, the link between individual differences in HRV and ventricular tachyarrhythmic risk in populations without known pre-existing cardiac conditions is less well explored. In this study we investigated the extent to which individual differences in resting state HRV predict susceptibility to spontaneous and pharmacologically-induced ventricular arrhythmias in healthy rats. Radiotelemetric transmitters were implanted in 42 adult male Wild-type Groningen rats. ECG signals were recorded during 24-h resting conditions and under β-adrenoceptor pharmacological stimulation with isoproterenol and analyzed by means of time- and frequency-domain indexes of HRV. No significant association was found between individual differences in resting measures of HRV and spontaneous incidence of ventricular arrhythmias. However, lower resting values of HRV predicted a higher number of ventricular ectopic beats following β-adrenergic pharmacological stimulation with isoproterenol (0.02 mg/kg). Moreover, after isoproterenol administration, one rat with low resting HRV developed sustained ventricular tachycardia that led to death. The present results might be indicative of the potential utility of HRV measures of resting cardiac autonomic function for the prediction of ventricular arrhythmias, particularly during conditions of strong sympathetic activation, in populations without known cardiac disease.

## 1. Introduction

Heart rate variability (HRV) refers to the beat to beat variation in the heart rate generated primarily by sympathetic and parasympathetic (vagal) influences on sinoatrial node activity. In humans, there are large individual differences in resting measures of HRV (e.g., reference [1]). A large body of experimental evidence indicates that several environmental factors, including lack of exercise [2], chronic stress [3], coffee consumption [4] and smoking [5], among others, can decrease HRV. However, it has now become clear that genetic factors determine 50% to 60% of observed individual differences in HRV measures in young adult humans [6,7]. The association between HRV and cardiovascular health has been the subject of much clinical research. For example, reduced HRV, reflecting impaired vagal tone and/or sympathetic hyperactivity [8,9], has been linked to modifiable and non-modifiable cardiovascular disease risk factors [10], although not without controversy [11]. Moreover, reduced HRV has been proposed as a prognostic marker of increased mortality and propensity to lethal ventricular arrhythmias in post-myocardial infarction and chronic heart failure patients [12,13,14,15,16]. It must be noted, however, that other studies have shown that increased HRV may also predict cardiac mortality particularly in elderly populations [17,18], suggesting a more complex interplay between patterns of autonomic nervous system activity and the electrical stability of the myocardium (e.g., reference [19]). Nevertheless, strong evidence in the literature suggests that conditions of elevated sympathetic and/or reduced vagal modulation increase susceptibility to lethal ventricular arrhythmia in cardiac diseases such as heart failure and prior myocardial infarct [20,21,22]. Therefore, the use of HRV indexes is an established risk stratification procedure in these patients [13,14]. In contrast, the association between HRV and the risk of cardiovascular events in individuals without known cardiovascular disease is far less well explored. A meta-analysis on this topic revealed that individuals with low HRV have ~40% increased risk of fatal or non-fatal cardiovascular events compared with individuals with high HRV [23], suggesting that HRV might also be useful in individual cardiac risk identification in a healthy group. However, the results of this meta-analysis should be interpreted with caution, given the small number of studies included and the inherent limitations of each individual study [23].

The investigation of the link between individual differences in HRV and risk of potentially life-threatening ventricular arrhythmias in a healthy population may be more easily addressed using animal models. For example, similar to what has been observed in humans, we have previously shown that several psychosocial factors (including chronic stress) can decrease resting HRV in rodents, with negative consequences on the electrical stability of the ventricular myocardium (reviewed in references [24,25]). Critically, if the relationship between individual differences in resting HRV and arrhythmia risk is confirmed in a non-selected population of healthy animals, this would pave the way for mechanistic investigations that are far more difficult to address in humans. In making this first observational step, it is worth highlighting a few considerations on the various indices of HRV, which is traditionally quantified by linear methods—time- and frequency-(spectral) domain analyses—both in humans and animals. In the time-domain, the root mean square of successive beat-to-beat interval differences (RMSSD) and, in the frequency-domain, the high frequency (HF) power component of HRV detect quick beat-to-beat fluctuations in a heart period time series, primarily reflecting vagal regulation of heart rate [26,27]. On the other hand, it has been theorized that the low frequency (LF) component of HRV represents an index of sympathetic outflow to the heart and that the LF/HF ratio reflects the state of the autonomic balance (e.g., reference [28]), although this stance is not without controversy. Specifically, more recent evidence suggests that the LF component is predominantly determined by vagal influences [29] and can also reflect other cardiac mechanisms such as baroreflex sensitivity [30] and the notion of sympathovagal balance has been disproven by a large body of research suggesting a more complex and often nonlinear relationship between the sympathetic and vagal components of the autonomic nervous system [31,32,33].

Therefore, given the generally accepted view of a protective role of the vagal input to the heart against vulnerability to ventricular arrhythmias [22,34], in this study we investigated the extent to which individual differences in resting state vagally-mediated HRV (indexed by RMSSD and HF) predict susceptibility to spontaneous and pharmacologically-induced ventricular arrhythmias in a non-selected population of adult male wild-type Groningen (WTG) rats. This outbred strain was selected to maximize phenotypic differences in HRV among individuals belonging to a genetically variable population.

## 2. Materials and Methods

### 2.1. Animals

Forty-two adult male wild-type Groningen (WTG) rats (Rattus norvegicus) were considered for this investigation, which was based on a secondary analysis of data from a previous study including 44 WTG rats [35]. Two rats were excluded from the present investigation as we could not retrieve their original ECG traces. The WTG strain was originally derived from the University of Groningen (The Netherlands) and bred in our department under conventionally clean conditions. Experimental procedures and protocols were approved by the University of Parma Animal Welfare Committee, in accordance with the European Community Council Directives of 22 September 2010 (2010/63/UE). Two weeks prior to surgery (see below), WTG rats were paired with an oviduct-ligated female partner in Plexiglas cages measuring 60 × 35 × 40 cm. Immediately after surgery, cages were divided transversely in two equal subzones (35 × 30 × 40 cm) by a wire mesh partition allowing olfactory, visual and acoustic contacts with the female partner, but no actual physical interaction. Rats remained confined to half of their housing cage throughout the experimental procedures described in the present investigation and were kept in rooms with controlled temperature (22 ± 2 °C) and lighting (lights on from 17:00 h to 05:00 h). Food and water were freely available and the bedding consisted of standard saw dust.

### 2.2. Surgery: Transmitter Implantation

At 15 weeks of age, rats were implanted, under tiletamine hydrochloride + zolazepam hydrochloride anesthesia (Zoletil Virbac, France, 20 mg/kg, s.c.), with radiotelemetric transmitters (TA11CTA-F40, Data Sciences International, St. Paul, MN, USA) for recordings of ECG (sampling frequency 1000 Hz) and locomotor activity (LOC, expressed as counts/minute) signals. The transmitter body was placed in the abdominal cavity; one electrode was fixed to the dorsal surface of the xyphoid process and another electrode was placed in the anterior mediastinum close to the right atrium, according to a previously described procedure [36]. This electrode location guarantees high-quality ECG recordings, even during vigorous physical activity. Subsequently, rats were prophylactically injected for 2 days with gentamicine sulfate (Aagent, Fatro, Italy, 0.2 mL/kg, s.c.) and allowed a 15-day recovery period.

### 2.3. 24-H Recording of ECG and LOC Signals

After recovery from surgery, ECG and LOC signals were recorded for 60 s every 60 min for ten consecutive days, with the animals left undisturbed in their home cages [35]. ECG and LOC signals were picked up by platform receivers (RPC-1, Data Sciences Int., St. Paul, MN, USA) and acquired via a ART-Gold 4.2 data acquisition system (Data Sciences Int., St. Paul, MN, USA).

### 2.4. β-Adrenoceptor Pharmacological Stimulation

The pharmacological challenge with isoproterenol occurred the day after the end of 24-h baseline recordings and was conducted before assigning rats to the two experimental conditions described in the original study [35]. On the day of the pharmacological challenge, baseline ECG tracings were recorded for 30 min, with the animals left undisturbed in their home cages. Rats were then injected with the β-adrenoceptor agonist isoproterenol (0.02 mg/kg, s.c., Sigma, St Louis, MO, USA) [37]. Isoproterenol was dissolved in saline solution (0.9% NaCl) and administered intraperitoneally in a volume of 0.5 mL/kg [37]. For each rat, ECG recording resumed immediately after isoproterenol injection and continued for 30 min in their home cages.

### 2.5. ECG Analysis

#### 2.5.1. Heart Rate and Heart Rate Variability

Initially, each raw ECG signal was visually inspected to ensure that all R-waves were correctly detected. Those parts of ECG recordings which exhibited recording artifacts were discarded without substitution and excluded from further analysis. Heart rate (HR; reported in beats per minute: bpm) and time- (i.e., RMSSD, ms) and frequency-domain (i.e., HF power, ms^2^; 0.75–2.5 Hz) parameters of vagally-mediated HRV were then quantified from 1-min ECG epochs using ChartPro 5.0 software (ADInstruments, Sydney, Australia). For spectral (frequency-domain) analysis of HRV, the power spectrum was obtained with a fast Fourier transform-based method (Welch’s periodogram: 256 points, 50% overlap and Hamming window). Mean HR and HRV values were calculated for each of the last three days of baseline recordings and then further averaged to obtain 24-h mean values. In addition, mean HR and HRV values were calculated for the 30-min pre-isoproterenol injection and 30-min post-isoproterenol injection periods.

#### 2.5.2. Quantification of Arrhythmic Events

**Figure 1:**
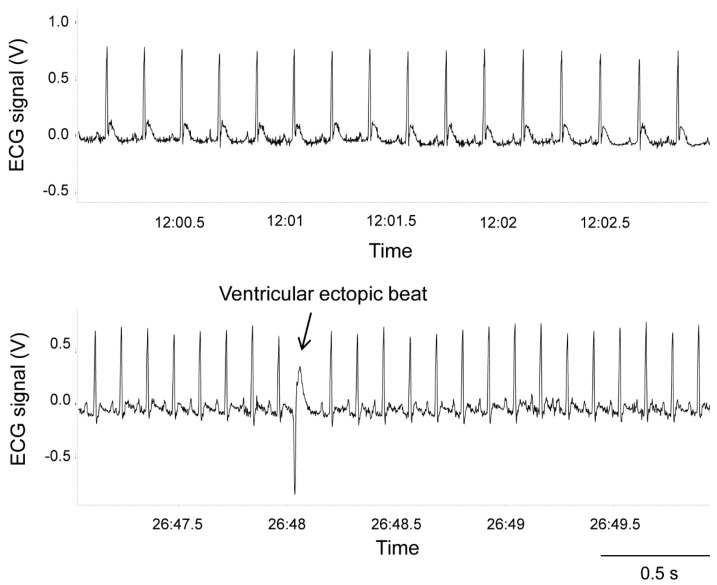
ECG tracings belonging to a representative wild-type Groningen rat with normal sinus rhythm (top panel) and isolated ventricular ectopic beat (bottom panel).

The occurrence of ventricular arrhythmias during baseline recordings (reported as number of events per length (hours) of an analyzed ECG recording) and the pharmacological challenge with isoproterenol (reported as the total number of events) was determined and quantified off-line based on the Lambeth Conventions (II) for the study of experimental arrhythmias [38] (Figure 1).

### 2.6. Statistical Analysis

**Figure 2:**
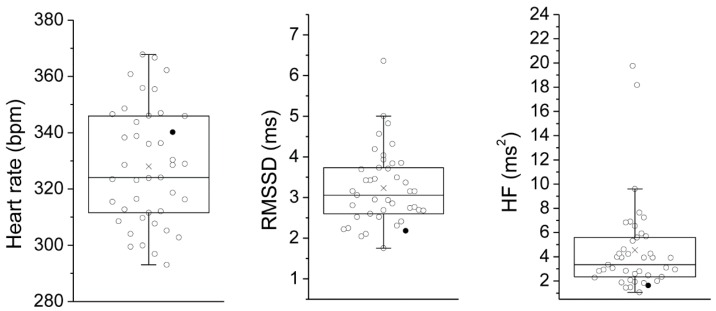
Box and whisker plots of the distribution of 24-h values of heart rate, RMSSD and HF in 42 wild-type Groningen rats. The empty circles indicate individual animals and the black circles indicate the rat that developed lethal ventricular tachycardia after isoproterenol administration. Circles beyond the whiskers represent outliers, according to the “1.5 × Inter-Quartile range rule”. RMSSD = root mean square of successive beat-to-beat interval differences; HF = high frequency.

All statistical analyses were performed using SPSS 24 software package (SPSS Inc., Chicago, IL, USA). Statistical significance was set at *p* < 0.05. Data were first checked for normality of variables and for violations of statistical assumptions of Linear Regression Models. As 24-h RMSSD and HF values were not normally distributed (Shapiro-Wilk test: *p* < 0.05 and *p* < 0.01, respectively) (Figure 2), these variables were transformed in their natural logarithms. Then, associations between 24-h values of HR, HRV and LOC and the incidence of spontaneous ventricular arrhythmias (SVA) were examined by Pearson correlations. Subsequently, to test cardiac autonomic and arrhythmogenic effects of isoproterenol injection, a series of paired *t*-tests was conducted on pre- and post-injection values. The median split of the 24-h values of HRV was used to divide the population into two groups, namely the “low HRV” and the “high HRV” groups. Then, the average number of ventricular arrhythmias following isoproterenol administration was compared between the two groups using an independent *t*-test. Lastly, a multiple hierarchical regression test was conducted to examine the relation between 24-h HRV values and the number of ventricular arrhythmias under β-adrenoreceptor pharmacological stimulation with isoproterenol, beyond the role played by SVA vulnerability. Step 1 (model 1) included the incidence of SVA during 24-h recordings as the predictor. Step 2 (model 2) added 24-h HRV values as the predictor.

## 3. Results and Discussion

As depicted in Figure 2, we found wide-ranging variance in 24-h measures of vagally-mediated HRV (RMSSD and HF values) in this sample of WTG rats (Figure 2). As expected, a significant inverse correlation was found between 24-h vagally-mediated HRV and HR values (Table 1). Critically, individual differences in cardiac autonomic parameters were not related to different levels of spontaneous somatomotor activity among individual rats (Table 1). This is an important confirmation that HRV measures represent trait-like individual differences in cardiac autonomic functioning in rats, similarly to what has been reported in both normal and clinical human populations in which resting states HRV indices show high test-retest reliability [39,40]. During resting conditions, animals showed a modest incidence of spontaneous ventricular arrhythmias, which did not correlate either with resting HR or vagally-mediated HRV (Table 1), suggesting that individual differences in cardiac autonomic parameters are not associated with differential electrical stability of the ventricular myocardium during resting conditions. In interpreting this finding, however, we wish to reiterate that the experimental animals were young adult, normal individuals with no documented cardiac conditions and therefore exhibited very few ventricular arrhythmias at rest, which may explain the lack of a significant association with cardiac autonomic parameters. 

In humans, normal aging is associated with changes in the autonomic control of sinoatrial node activity. Specifically, a progressive impairment in cardiac parasympathetic influences, which is reflected by increased resting HR and reduced HRV, has been observed with age [41,42]. This decline in vagal control of cardiac function has been ascribed to a deterioration of vagal baroreflex sensitivity and is thought to contribute to increased risk of arrhythmias and sudden cardiac death in elderly populations [43,44]. Likewise, in a previous study in this rat strain we found a clear decrease in vagally-mediated HRV (HF values) during the final stage of the aging process [45]. Remarkably, this vagal impairment was associated with an increase in the number and complexity of spontaneous arrhythmic events [45]. Taken together, these findings indicate that while individual differences in resting measures of HRV may not be useful for ventricular arrhythmia risk stratification during unchallenged conditions in young populations, they might be a critical factor to consider for early detection of individual susceptibility to ventricular arrhythmias in aged hearts [46].

**Figure 3:**
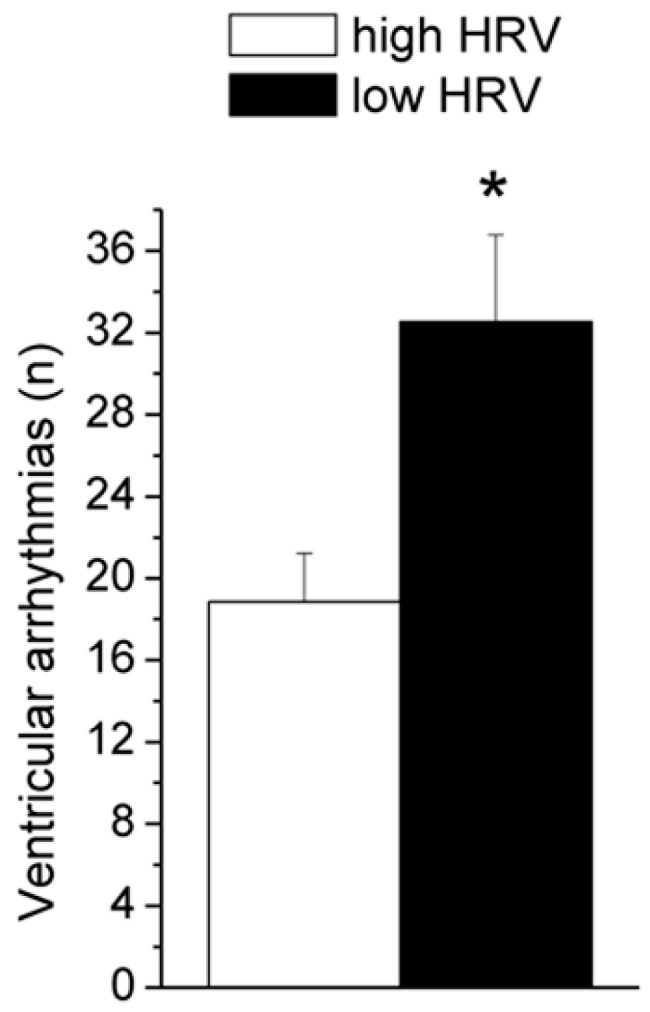
Number of ventricular arrhythmias following administration of isoproterenol in the low HRV (n = 20) and high HRV (n = 21) groups. Data are expressed as mean ± SEM. The median split of lnRMSSD values (1.15) during 24-h recordings was used to divide the population into these two groups. The rat that died shortly after isoproterenol administration was not included in these analyses. HRV = heart rate variability; lnRMSSD = natural logarithm of the root mean square of successive beat-to-beat interval differences; * = *p* < 0.05.

**Figure 4:**
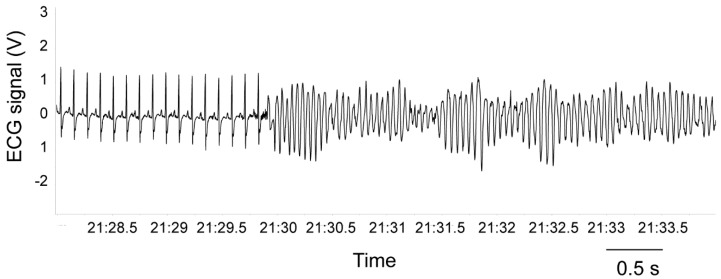
ECG tracing before rat’s death with sustained ventricular tachycardia after administration of the β-adrenergic agonist isoproterenol.

As reported in Table 2, β-adrenergic pharmacological stimulation with isoproterenol provoked a potent tachycardic response and a large increase in the incidence of ventricular ectopic beats. Importantly, the number of ventricular arrhythmias following isoproterenol injection was significantly higher in rats with low 24-h HRV values compared to rats with high 24-h HRV values (*t* = 2.4, *p* < 0.05) (Figure 3). Critically, vulnerability to spontaneous ventricular arrhythmias (as assessed during 24-h ECG recordings) was not a significant predictor of the number of ventricular ectopic beats under β-adrenoreceptor pharmacological stimulation with isoproterenol (Table 3, Model 1). However, the model including the vagal index RMSSD significantly added to the prediction, explaining an additional 10% of the variance, with R-squared for the total model being 0.10 (Table 3, Model 2). In other words, lower resting values of vagally-mediated HRV predicted a higher number of ventricular arrhythmic events following β-adrenergic pharmacological stimulation with isoproterenol (r^2^ = 0.10). Because lnRMSSD and lnHF-HRV were strongly correlated (Table 1), only lnRMSSD was used in these analyses as it is thought to be less affected by respiratory influences [27], as well as in light of the observation that time domain parameters of HRV are estimated with smaller bias and variability compared with frequency domain parameters [47]. Results, however, do not change when RMSSD is replaced with HF-HRV. Although ventricular ectopic beats are usually considered as being benign in asymptomatic healthy subjects [48], some studies have shown that they may be associated with adverse cardiovascular outcomes [49,50]. Specifically, many cases of sudden death in post-myocardial infarction and chronic heart failure patients are thought to result from ventricular tachyarrhythmias [20,21]. Importantly, in one rat of the present study, the injection of the β-adrenergic receptor agonist provoked sustained ventricular tachycardia that led to death (Figure 4). Remarkably, this rat was characterized by very low values of vagally-mediated HRV during resting conditions (i.e., lower quartile of the distribution; Figure 2), suggesting that low resting vagal modulation of cardiac function may be associated with a higher risk for potentially malignant ventricular tachyarrhythmias in individuals without known cardiovascular disease under conditions of potent sympathetic stimulation. Supporting this view, in a previous study we reported a similar case of isoproterenol-induced lethal ventricular tachycardia in a WTG rat belonging to a group of animals selected for high levels of trait aggressiveness and characterized by lower resting values of vagally-mediated HRV compared with the non-aggressive group [51].

The effect of sympathetic stimulation on global ventricular electrophysiology has been studied extensively in animal models [52,53] and humans [54], although the precise mechanisms still need to be clarified [22]. Particularly relevant for the present study are previous findings from our group showing reduced refractoriness and impaired conduction (i.e., major determinant of arrhythmogenesis) in the left ventricle of WTG rats after repeated, stress-induced activation of the sympathetic nervous system [55]. On the other hand, vagal nerve stimulation has been shown not only to slow down the sinus rate but also to display many beneficial effects that protect the heart against ventricular arrhythmias [22], with the mechanistic basis that may involve effects both on sympathetic signaling and on ventricular electrophysiology itself (reviewed in reference [56]). Indeed, the historical belief that parasympathetic innervation of the ventricular system is sparse and parasympathetic control of the ventricular contractility is insignificant has been challenged by significant evidence obtained in various species (from rats to humans) demonstrating the presence of choline acetyltransferase-positive nerve fibers, acetylcholinesterase enzyme and muscarinic receptors in both the right and left ventricles (reviewed in reference [57]). Moreover, functional data clearly demonstrate that increased activity of the vagus nerve decreases the force of ventricular contraction independent of its effect on HR [58].

## 4. Conclusions

The present results demonstrate the existence of a negative relationship between HRV measures of resting vagal tone and vulnerability to ventricular arrhythmias under β-adrenoreceptor pharmacological stimulation in a non-selected population of young adult rats with no documented cardiac conditions. This might be indicative of the utility of HRV measures of resting cardiac autonomic functioning for the prediction of potentially life-threatening ventricular arrhythmias not only in the clinical setting, but also in normal populations. It must be acknowledged, however, that the episode of sudden death that occurred in one animal with low resting HRV remains, to some extent, anecdotal and particular caution must be taken in interpreting these findings. It will thus be important to replicate these results in other species of rats and/or older rats. However, the present results may provide the basis for future studies, using non-selected populations of rats with large individual differences in vagally-mediated HRV, to investigate the pathophysiology that underlies the generation of ventricular arrhythmias and explore whether adverse remodeling of the heart can be revealed prior to the onset of the cardiac disease phenotype in normal individuals with low resting HRV.

## Figures and Tables

**Table 1:** Correlation matrix of 24-h cardiac autonomic and locomotor parameters in 42 wild-type Groningen rats.

	Mean (SEM)	HR	lnRMSSD	lnHF	SVA	LOC
HR	328 (3)	-				
lnRMSSD	1.14 (0.04)	−0.38 *	-			
lnHF	1.30 (0.10)	−0.41 *	0.98 *	-		
SVA	0.49 (0.11)	0.19	0.09	0.05	-	
LOC	4.52 (0.12)	0.23	−0.01	−0.05	0.09	-

Notes. HR = heart rate (reported in beats/minute); lnRMSSD = natural logarithm of the root mean square of successive beat-to-beat interval differences; lnHF = natural logarithm of the high frequency power; SVA = spontaneous ventricular arrhythmias (reported in the number of events per length (hours) of analyzed ECG recording); LOC = locomotor activity (reported in counts/minute). * *p* < 0.05.

**Table 2:** Heart rate and lnRMSSD values and number of ventricular arrhythmias before and after the injection of the β-adrenergic agonist isoproterenol in 41 wild-type Groningen rats.

	Pre-Isoproterenol	Post-Isoproterenol
Heart rate (beats/minute)	358 ± 7	485 ± 3 *
lnRMSSD	0.86 ± 0.05	0.64 ± 0.05 *
Ventricular arrhythmias (n)	0.3 ± 0.1	25.9 ± 2.7 *

Data are expressed as mean ± SEM of the 30-min pre-isoproterenol and post-isoproterenol recording periods. The rat that died short after isoproterenol administration was not included in these analyses. lnRMSSD = natural logarithm of the root mean square of successive beat-to-beat interval differences; * = *p* < 0.05 vs. the corresponding pre-injection period.

**Table 3:** Summary of the hierarchical regression analysis for variables predicting the total number of ventricular arrhythmias under β-adrenoreceptor pharmacological stimulation with isoproterenol.

**Model 1**	***B***	***SE***	**β**	**95%CI**
Constant	27.48	5.03		
SVA	−0.88	2.39	−0.06	−5.71; 3.96
			r^2^ = 0.00	
**Model 2**	***B***	***SE***	**β**	**95%CI**
Constant	51.44	12.57		
SVA	−1.25	2.30	−0.83	−5.91; 3.42
lnRMSSD	−20.36	9.86	−0.32 *	−40.31; −0.40
			r^2^ = 0.10	

Notes. *B* = unstandardized coefficient; *SE* = standard error; β = standardized coefficient; CI= confidence interval; VIF = variance inflation factor; SVA = spontaneous ventricular arrhythmias; lnRMSSD = natural logarithm of the root mean square of successive beat-to-beat interval differences. * *p* < 0.05. The rat that died shortly after isoproterenol administration was not included in these analyses.
